# 1799. Patients Who Inject Drugs (PWID) and Unstable Housing are Associated with Invasive Group A Strep Infection: A retrospective collaboration between two large, quaternary care hospital systems in Philadelphia

**DOI:** 10.1093/ofid/ofad500.1628

**Published:** 2023-11-27

**Authors:** Nicole Loeven, Eric Altneu, Stephanie Spivack, Virginie Halpern-Cohen, Kelly Dyer, Kaede Ota Sullivan, Shara Epstein, Daniel Teixera da Silva, Jessica Meisner, Sara K Schultz

**Affiliations:** University of Pennsylvania Perelman School of Medicine, Philadelphia, Pennsylvania; Temple University Health System, Philadelphia, Pennsylvania; Temple University Health System, Philadelphia, Pennsylvania; University of Pennsylvania Perelman School of Medicine, Philadelphia, Pennsylvania; University of Pennsylvania Perelman School of Medicine, Philadelphia, Pennsylvania; Temple University Health System, Philadelphia, Pennsylvania; Philadelphia Department of Public Health, Philadelphia, Pennsylvania; Philadelphia Department of Public Health, Philadelphia, Pennsylvania; University of Pennsylvania Perelman School of Medicine, Philadelphia, Pennsylvania; Temple University Hospital, Philadelphia, Pennsylvania

## Abstract

**Background:**

Invasive infections involving Group A *Streptococcus* (iGAS), defined as infections beyond simple skin and soft tissue infection or pharyngitis, are rising nationally. We report an increase in incidence of iGAS at two urban, quaternary care health systems that contain 6 hospitals in the city of Philadelphia.

**Methods:**

All blood, sterile fluid, and tissue cultures that yielded *Streptococcus pyogenes* were identified using the laboratory information systems at the Temple University Health System and the University of Pennsylvania Health System. Two cohorts were compared: cases diagnosed between January 1, 2019 and December 31, 2019, and between January 1, 2021 to December 31, 2021. We excluded 2020 data to avoid analysis of disease incidence when hospital admission patterns may have been highly biased due to COVID-19 pandemic. Electronic health records were reviewed and data pertaining to demographics, month of infection, culture site, HIV status, HCV status, housing status, and injection drug use were abstracted. Descriptive statistics were used to summarize findings.

**Results:**

In 2021, there were 301 unique cases of iGAS (148 involving bacteremia) compared to 169 in 2019 (with 70 cases of bacteremia). This represented an overall increase in iGAS of 178% and a 211% increase in cases involving bacteremia. Of the cases in 2021, 210 (69.8%) were PWID compared to only 54 (32%) in 2019, a 388% increase. In 2021, 7.5% of patients had a diagnosis of HIV compared to 8.9% in 2019, but a large percentage did not have an HIV test (37% in 2019 and 19% in 2021). HCV Antibody positivity was 52.3% in 2021 versus 26.6% in 2019. Housing instability was recorded in 151 cases (50.2%) in 2021 and 31 (18.3%) in 2019, a 487% increase.

Table 1

**Figure:**
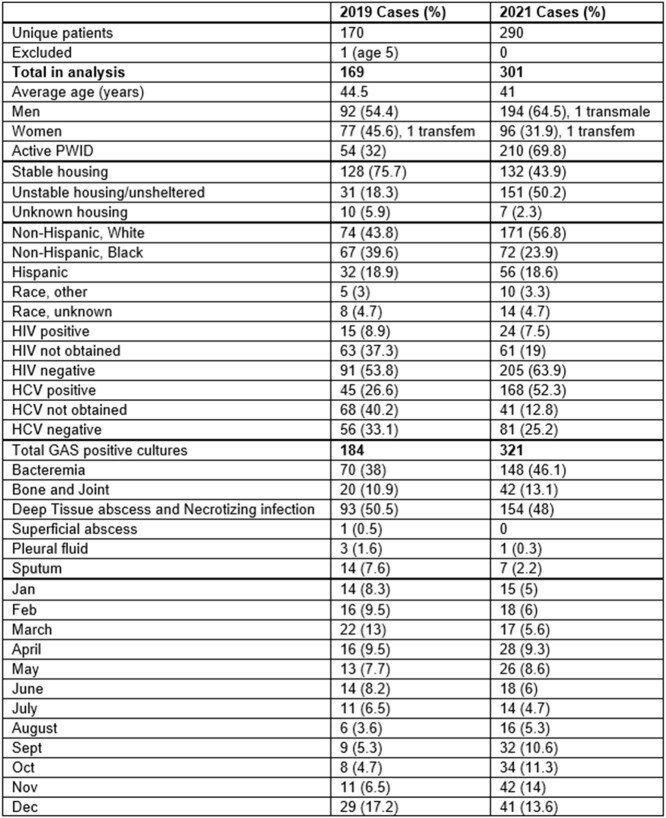

Demographic and clinical characteristics of iGAS cases in 2019 and 2021

Table 2

**Figure:**
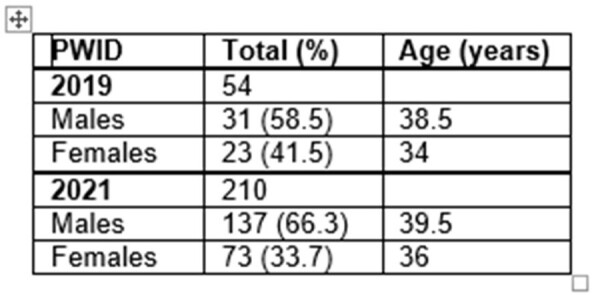

Age and Sex data in PWID

**Conclusion:**

From 2019 to 2021, there was an increase in iGAS infections among an adult population in a large metropolitan city. We noted a 178% total increase in iGAS during our study period, with a 388% increase among PWID and 487% increase among those unstably housed. As the overdose crisis continues, public health practitioners and medical providers should be aware of the increase in iGAS when providing empiric treatment for PWID with infections and utilize local and national resources to report and manage cases.

**Disclosures:**

**Sara K. Schultz, MD FACP FIDSA**, AbbVie: Advisor/Consultant

